# Helminth Communities of Common Fish Species in the Coastal Zone off Crimea: Species Composition, Diversity, and Structure

**DOI:** 10.3390/biology12030478

**Published:** 2023-03-21

**Authors:** Mariana P. Plaksina, Evgenija V. Dmitrieva, Alexander G. Dvoretsky

**Affiliations:** 1 Murmansk Marine Biological Institute of the Russian Academy of Sciences (MMBI RAS), 183010 Murmansk, Russia; 2 A.O. Kovalevsky Institute of Biology of the Southern Seas, 119991 Moscow, Russia

**Keywords:** helminths, fish parasite, Black Sea, infracommunity, component community, nestedness

## Abstract

**Simple Summary:**

Despite a long research history of fish parasites in the Azov–Black Sea region, the helminth community structure among fish populations remains poorly understood. Until recently, only a few articles considering the helminth communities of mullet fish from this region have been published. In the present paper, the data on the species diversity and the structure of the helminth communities of 12 common fish species are presented for coastal waters off Crimea. These communities are characterized by low species richness and relatively low diversity indices. Component communities are more diverse than infracommunities. Although these parameters correspond to unbalanced, immature communities, the modern helminth communities of Crimean marine fish are well-aggregated and follow a “core–satellite” distribution, indicating their balanced structure. There are no negative inter-specific relationships in most of the component communities. Obtained data expand the current knowledge regarding the organization of parasite communities in the region and may have important implications for the further monitoring and proper management of local fish stocks.

**Abstract:**

In this paper, we analyzed the diversity and structure of helminth communities of 12 common fish species from the coastal zone of Crimea. A total of 53 helminth species were found. The total number of parasite species per host fish ranged from 3 to 18. Species richness at the infracommunity and component community levels were from 1.4–4.2 to 1.7–7, respectively. The Brillouin index for the infracommunites was 0.1–1, while the Shannon index for the component communities was 0.3–1.2. Component communities demonstrated a bi- or tri-modal distribution of the parasite prevalence and positive correlations between the prevalence and log-transformed abundance indices, thus following the “core–satellite” conception. Overall, the prevalence and abundance index of the dominant parasite in the component communities ranged from 18 to 80% and from 0.6 to 61.5 ind. per fish, respectively. The structure of the helminth component communities demonstrated good accordance with the nestedness mode where the rarest species occurred in the most diverse infracommunities, while the poorest infracommunities were composed of a few dominating species. More than two-thirds of the studied helminth species had an aggregated distribution indicating well-structured and developed communities. Our data provide a basis for further research and may be used for fish resource monitoring and management.

## 1. Introduction

The helminth fish fauna is being recognized as an important field for parasitological research because of their major impact on the fish industry [1,2,3,4,5]. Several helminth species are known to affect the growth, reproduction, and survival of the hosts [6,7] and cause morbidity and mortality, thus reducing important fish stocks [8,9]. Some of these infections have zoonotic potential when humans consume raw or undercooked fish containing infective parasite larvae [3,5,10,11].

Helminths have been known and studied since ancient times, but the concept of them as objects of independent ecological studies, as members of biocenoses, began to form less than 100 years ago [12,13,14]. Two comprehensive monographs by Dogiel [15,16] presented basic information on the dependence of the parasitocoenosis (i.e., the totality of all parasites in a host) on the environment and discussed the role of biological and physical factors in driving the assemblages of parasitic species associated with some free-living organisms or biocenoses. During this period, ecological studies of parasites have advanced considerably [17]. Various schemes for classifying parasite association have been proposed, but the best known and widely used is that of Bush et al. [18], who proposed the following gradation for parasitic communities: (a) all organisms simultaneously occurring in a single host specimen constitute an infra-community; (b) all infra-communities in hosts of the same species existing simultaneously at the same location constitute a component community; (c) the aggregate of all component communities including free-living stages of the parasite life cycle in a given ecosystem form a composite community. Inter-specific relationships are not a prerequisite for these systems [19,20,21]. Thus, component parasite communities are subsets of the parasite fauna of a host species and these communities often form saturated communities, such that their richness is not always a reflection of that of the entire parasite fauna. The number of species in a component community is instead influenced by the local availability of parasite species, probability of colonization, and parasite specialization. At the lowest level, infracommunities in individual hosts are subsets of the species occurring in the component community [21].

Since the 1960s, numerical approaches to studying fish parasite communities have replaced descriptive studies, which mainly considered component communities. In turn, many papers have been aimed at studying the ecological factors and processes in the communities and the relationships between parasites within the same host or population [22,23,24,25,26,27,28,29]. As a result, classical diversity indices (Shannon, Pelou’s evenness, Berger–Parker, and others) have been involved in parasitological studies [30,31,32,33,34]. Helminth communities have been shown to vary spatially and temporally [35,36] depending on historical, biogeographical, and ecological factors [37,38], among which the total abundance and, therefore, the availability of potential hosts, has been recognized as being the most important [39,40]. Other significant factors driving fish parasite faunas are characteristics of the locality, predator–prey relationships, relationships between different phylogenetic groups of parasites, and stochastic processes across multiple scales and intensities [41]. The importance of the host size has been confirmed in many studies [42,43,44,45,46,47,48], although others have revealed no clear relationships with the host size [17,49].

Nestedness is well-described for parasites and has been widely used to test the non-random structure of their component communities [7,44,50,51,52,53,54,55,56,57,58,59,60,61]. Patterson and Atmar [62] proposed a method to explore various features of nestedness including causation. This separate analysis is used to evaluate each potential determinant in insular systems that exhibits a pattern of species composition termed “nested subsets”, in which the species comprising a small fauna or flora represent a proper or included subset of those on larger, richer systems, rather than a random draw of those found in the entire species pool [63,64,65].

The majority of studies have revealed a significant level of nestedness in the parasite component communities studied, but factors determining the formation of such a structure are not fully understood.

Despite the extensive literature on surveys of fish parasites and taxonomic studies (see, e.g., an overview in [66]), the structure of infracommunities and component communities of fish parasites in the Azov–Black Sea region has not yet been well-described, although local habitats are diverse and complex and provide excellent conditions for spawning and juvenile fish and shellfish survival and development, supporting abundant populations of commercially important species [67,68]. Pankov [69] assessed diversity indices of helminths in four species of the family Mugilidae from Bulgarian waters of the Black Sea. A number of papers analyzed parasite communities in the grey mullet *Mugil cephalus* and the invasive redlip mullet *Planiliza haematocheilus* [70,71,72,73,74,75,76,77,78].

Taking into account the potential impact of fish parasites on fish productivity and their importance for the fish industry, the knowledge of helminth infection both at the individual fish level and in fish stocks is necessary to understand the parasite distribution patterns and infection processes in the area. Moreover, these data may be important to determine the population structure and migration patterns of the hosts [79], and therefore, for the proper management of local fish stocks.

Our aim was to provide a general description of the diversity, structure, and interspecific relationships of helminths in infra- and component communities in selected abundant and commercially important fish species from the coastal zone of Crimea.

## 2. Material and Methods

### 2.1. Study Area and Study Objects

The Black Sea is considered as a semi-enclosed basin of the Mediterranean Sea. The main features of the sea are a sharp halocline at 50–100 m induced by the inflow of salty Mediterranean waters in the deeper layers, and the large riverine inflow at the surface layers [80] and strong stratification, promoting the presence of the anoxic and almost lifeless conditions in the Black Sea deep layers below 100–200 m [81]. The Sea of Azov is a shallow (depth 0.9–14 m) inland sea connected to the Black Sea by the narrow Strait of Kerch (4 km wide).

**Figure 1:**
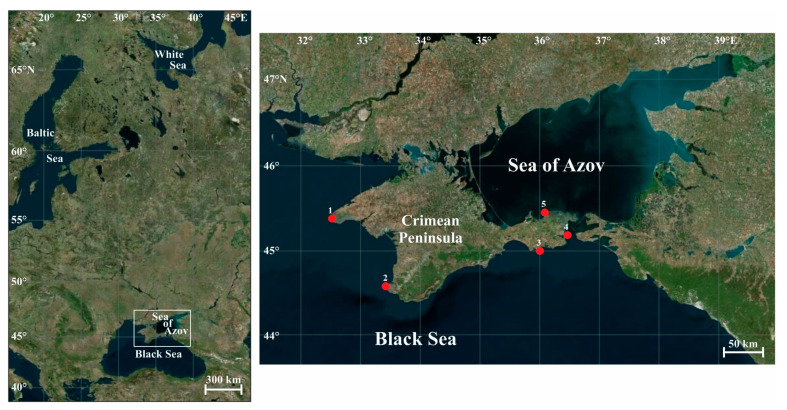
Map of the study area and sampling locations, 2009–2016. 1—Cape Tarkhankut (45.33° N, 32.47° E); 2—Sevastopol (44.60° N, 33.43° E); 3—Karadag Nature Reserve (45.13° N, 35.19° E); 4—Kerch Strait (45.13° N, 34.42° E); 5—Cape Kazantip (45.47° N, 35.83° E).

The Crimean Peninsula is situated on the 45th parallel (Figure 1), in the middle between the equator and the pole, on the border between the temperate and subtropical climate zones, and is washed by waters of the Black Sea and the Sea of Azov [67].

The peninsula has a cruciform shape extending about 180 km to the south from the mainland and 360 km from the west to the east. In winter, the northern part of the area may be ice-covered, whereas, in the southern part, water temperatures are usually higher than 6 °C. In summer, the water temperature may reach 30 °C, but usually 26 °C [82].

Of the fish taxa selected (Table 1), nine species are of commercial importance, namely, *Engraulis encrasicolus*, *Trachurus mediterraneus*, *Chelon auratus*, *Mugil cephalus*, *Belone belone*, *Atherina boyeri*, *Atherina hepsetus*, *Spicara smaris*, and *Planiliza haematocheilus*. The latter species has been introduced in the Azov–Black Sea region [83].

### 2.2. Parasitological Analysis

Fishes were caught by commercial fishermen during the period 2009–2016. Fresh specimens were dissected and examined under a stereomicroscope. Helminths were collected, preserved, and stained according to standard methods [84,85]. Parasites were identified under an Olympus C41 microscope (Olympus Corporation, Tokyo, Japan) with ×100–×2000 magnification and a phase-contrast device using published guides [86] and recent taxonomic revisions of some helminth groups (e.g., [87,88,89,90,91]).

The prevalence (P) and mean abundance (AI) were calculated as per Bush et al. [18]. Data analysis was carried out at the infracommunity and component community levels. An infracommunity is a community of the parasite in a single host individual and a component community refers to all parasites in one sample of hosts belonging to one species and collected simultaneously sensu Bush et al. [18]. Only those component communities with at least 10 infracommunities (i.e., infected fish per sample) were included in the analysis.

Although protozoa and myxosporidia were also found during the parasitological examination, they were not included in the analysis as there are no methods that would allow for a count of their exact number in the infracommunities. In addition, two parasitic crustaceans were found (*Clavallisa emarginata* and *Ergasilus nanus*), but their prevalence and abundance were very low. For these reasons, we only focused on helminths.

### 2.3. Estimation of Community Diversity and Structure

The following diversity indices were calculated for each community: N—species richness; HB—Brillouin index for infracommunity; H—Shannon index for component community; J—Pielou’s evenness; and d—Berger–Parker dominance index [34,92]. The Spearman rank correlation coefficient was used to estimate the correlation between the diversity, dominance, and evenness indices of infracommunities and the corresponding characteristics of the component communities they comprise.

According to Hanski [93], “if stochastic variation in the rates of local extinction and/or colonization is sufficiently large, species tend to fall into two distinct types, termed the “core” and the “satellite” species. The former is regionally common and locally abundant, and relatively well spaced-out in niche space, while opposite attributes characterize satellite species”. To test the possible existence of such “core” and “satellite” species in the helminth communities examined, a modal analysis of the frequency distributions was conducted and a correlation coefficient between the mean abundance (AI) of a helminth and its prevalence (P) was calculated for each host species. In addition, cluster analysis of P and AI data was carried out on the basis of Euclidean distances using the complete linkage method.

The NODF index, a nestedness metric based on the overlap and decreasing fill, proposed by Almeida-Neto et al. [94], was calculated to study the structure of helminth communities. NODF = ΣNODF_paired_/[s(s − 1)/2] + [m(m − 1)/2], where s is the number of species, m is the number of sites (fish specimens), and NODF_paired_ is the pairwise degree of nesting (i.e., counting of how many interrelated pairs of species and how many interrelated pairs of sites (fish) exist in particular subsets of matrix elements). NODF ranges from 0 (different species do not occur in the same community) to 100 (each fewer common species co-occurs only with more common species). The null hypothesis (H_0_) for the stochastic nature of the species distribution was performed based on the CE randomization model, which generates matrices with row and column sums proportional to the row and column totals of the original matrix [95]. The number of generated null matrices was 999. The comparison of the nestedness value of the matrix under examination with those of a set of null matrices was carried out using a Z-value calculated as [NODFobs − m(NODFgen)]/SD(NODFgen), where NODFobs is the nestedness of the matrix under study, and m(NODFgen) and SD(NODFgen) are, respectively, the mean and standard deviation of the nestedness values of the null matrices. The standardized z-score of >1.64 indicates that the degree of nestedness is not random at a 95% significance level. NODF index calculations, null model generations, Z statistics, and the visualization of nestedness were carried out in NeD software [95].

To assess the level of parasite aggregation in the component communities, the coefficient *b* of the Taylor power-law was calculated for the following equation: log(S^2^) = log(*a*) + *b* × log(AI), where s^2^ is the variance; AI is the abundance index; *b* is the index of heterogeneity: *b* > 1 indicates an aggregated distribution, *b* = 1 indicates a random distribution, and *b* < 1 indicates a uniform distribution [96,97]. These relationships were studied for 31 of the 53 helminth species to ensure a sample size of 10 fish specimens or more infected by each parasite, and provide at least five such component communities. The Student t-test was applied to reveal if the *b*-value differed significantly from 1. A significance level was set at α = 0.05.

Diversity indices and graphs were calculated and plotted in Past3 [91].

## 3. Results

### 3.1. Diversity of Infra- and Component Communities

**Figure 2:**
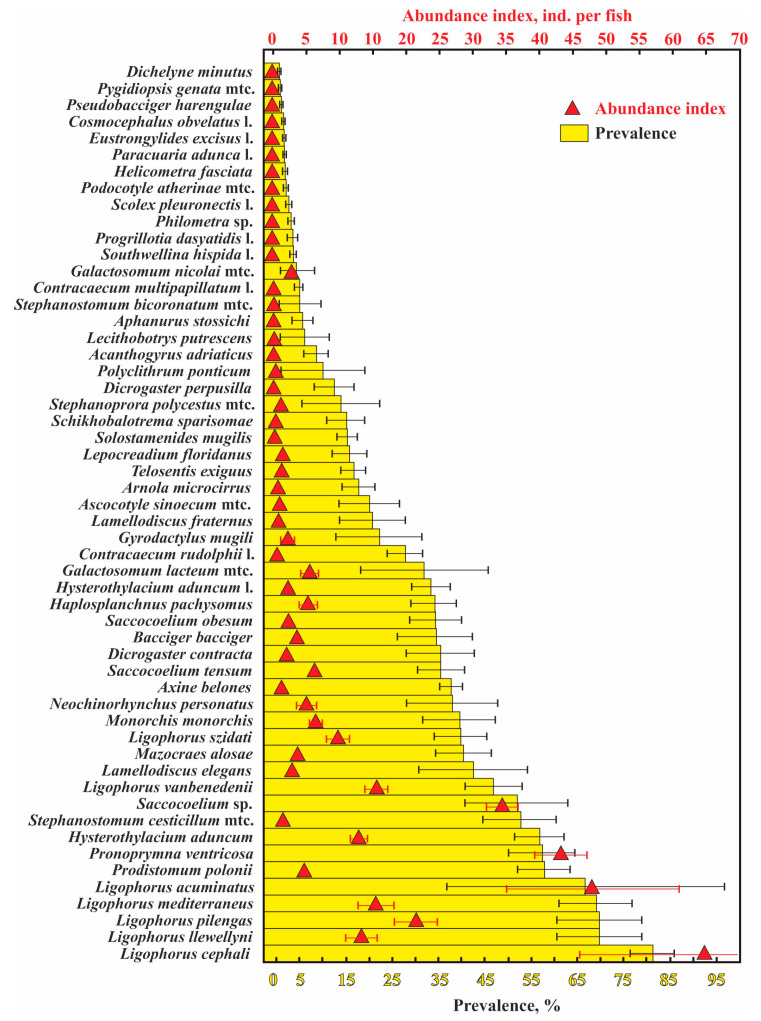
Mean values (±standard errors) for the abundance index and prevalence of infection of helminthes in common fish species from waters near the Crimean Peninsula. Note that the infection indices for *Hysterothylacium aduncum* are presented separately for definitive and intermediate hosts. Infection indices for communities only (i.e., without unparasitized fish specimens) are presented.

A total of 53 helminth species were found in the 12 examined fish species; 2211 infracommunities (=number of fish infected) and 103 component communities (number of fish samples, in which at least 10 specimens were infected) (Table 1). The most common and abundant parasites were monogeneans of the genus *Ligophorus*. The majority (11 of 15 species) of the least numerous helminth species, with a prevalence ≤5% and an abundance index ≤0.1 ind. per fish, were at their larval stage (Figure 2).

In contrast to other groups, monogeneans occupied all the studied component communities in which they could be found (Table 2).

In general, the highest number of parasites was registered in the golden grey mullet *Chelon auratus*, sand smelt *Atherina boyeri*, and grey mullet *Mugil cephalus*. Members of the family Mugilidae were infected by specialist parasites (20–30%) and intermediate generalists (i.e., parasites which are species specific to the fish family (20–30%), while both species of sand smelt (*Atherina* spp.) were parasitized only by true generalists (i.e., parasites with a wide range of host species from different families) (Table 1).

Infracommunities of fish parasites in the Crimean waters had low diversity indices (Table 3).

The maximum Brillouin index (HB) was 1.7, but for the majority of fish species, the mean HB did not exceed 0.5 and the Berger–Parker dominance index for these communities was 0.8–0.9. The highest values of HB and the Pielou evenness index were registered for golden grey mullet, redlip mullet, and grey mullet. No significant correlation was revealed between the total number of species found in each fish species (Table 1) and HB as well as between the average species richness in the infracommunities and HB (Table 3): r-Spearman was 0.50 and 0.44, respectively, at *p* > 0.05. For example, sand smelt were parasitized by 15 species, but the number of species per infracommunity did not exceed 5, whereas, in golden grey mullet and grey mullet, which have similar numbers of parasites (18 and 12, respectively), the number of species per infracommunity and HB was 2 and 5 times higher than in the sand melt *Atherina boyeri*. Redlip mullet with eight parasite species had a HB equal to the golden grey mullet but 3 times higher than in the sand melt. These patterns may be explained by high proportions of specialist species (17–33%) with high infestation indices in the mugilid species (Table 1 and Table 2). In contrast, the helminths of sand smelt were generalists (Table 1), half of which are typical parasites of freshwater and brackish water fish species and, therefore, they were incidental parasites for *Atherina boyeri*. The high values of the Berger–Parker dominance index (0.5–0.9) were attributable to the presence of 1–2 species in the majority of infracommunities (73%).

Analysis of component communities indicated that their diversity indices were low, albeit slightly higher than in the infracommunities (Table 4).

The highest Shannon indices (0.90–1.20) were found for the parasite component communities of horse mackerel, two sand smelt species, golden grey mullet, and grey mullet. At the same time, the component communities of *Atherina boyeri*, *A. hepsetus*, and *Trachurus mediterraneus* demonstrated significantly higher values of species richness and Pielou’s evenness than their infracommunities. However, in general, diversity indices of the component communities and their constituent infracommunities were positively correlated with each other. The r-Spearman was 0.66 for the correlation between the numbers of species in the infra- and component communities; 0.53 for the HB vs. H; 0.60 and 0.53 between the J and d indices in the infra- vs. component communities, respectively, at a significance level of *p* < 0.05 for all correlations.

### 3.2. Structure of Component Communities in View of the “Core–Satellite Species” Hypothesis

**Figure 3:**
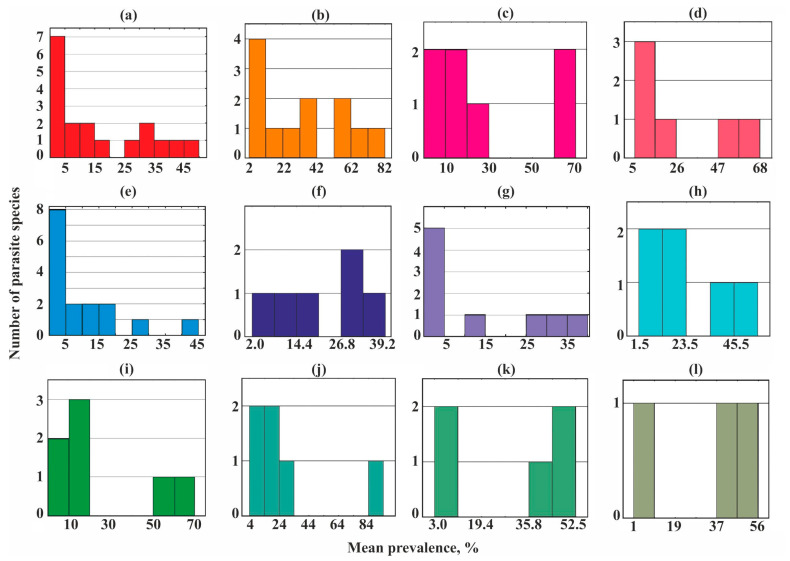
Frequency distribution of parasite prevalence in common fish species from Crimea. (**a**)—*Chelon auratus*; (**b**)—*Mugil cephalus*; (**c**)—*Planiliza haematocheilus*; (**d**)—*Chelon saliens*; (**e**)—*Atherina boyeri*; (**f**)—*Atherina hepsetus*; (**g**)—*Belone belone*; (**h**)—*Diplodus annularis*; (**i**)—*Trachurus mediterraneus*; (**j**)—*Spicara smaris*; (**k**)—*Alosa immaculata*; (**l**)—*Engraulis encrasicolus*.

A bimodal distribution occurred in most helminth communities studied (Figure 3) while three groups of species (core, satellite, and rare) were revealed in the parasite communities of the sand smelt *Atherina boyeri* and garfish *Belone belone* (Figure 3e,g).

**Figure 4:**
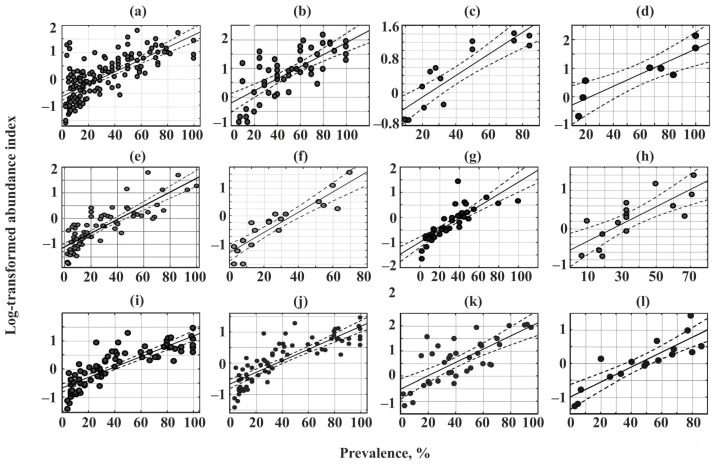
Relationships between the prevalence and log-transformed abundance indices of parasites in common fish species from Crimea. (**a**)—*Chelon auratus*; (**b**)—*Mugil cephalus*; (**c**)—*Planiliza haematocheilus*; (**d**)—*Chelon saliens*; (**e**)—*Atherina boyeri*; (**f**)—*Atherina hepsetus*; (**g**)—*Belone belone*; (**h**)—*Diplodus annularis*; (**i**)—*Trachurus mediterraneus*; (**j**)—*Spicara smaris*; (**k**)—*Alosa immaculata*; (**l**)—*Engraulis encrasicolus*.

A significant positive correlation between the prevalence and log-transformed abundance index was found for all communities (Figure 4, Table 5).

**Figure 5:**
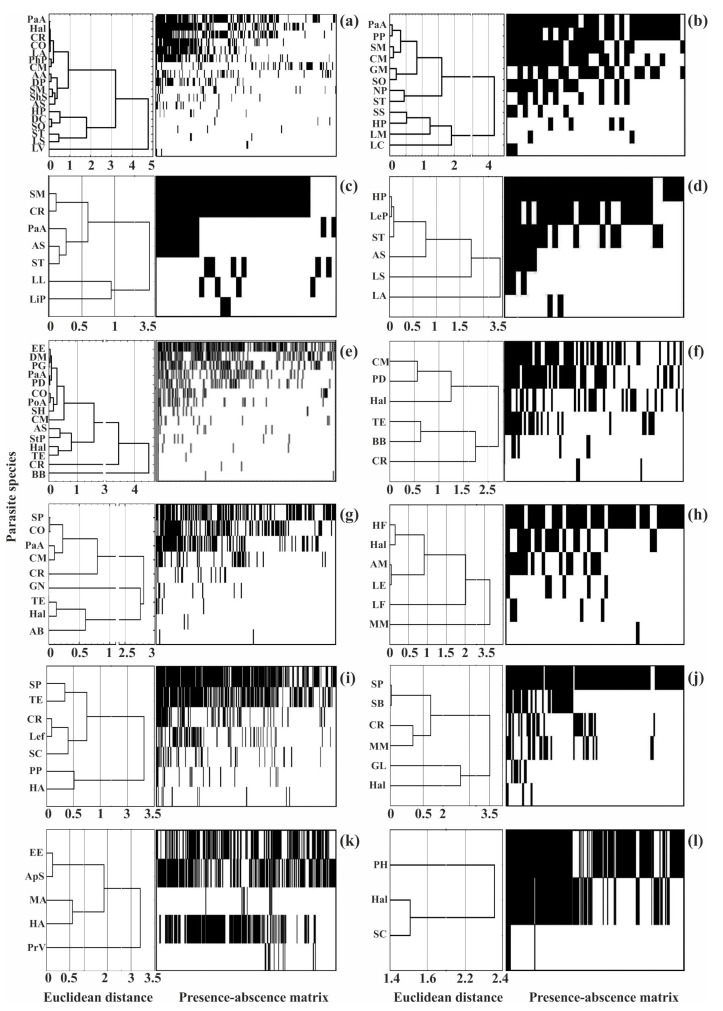
Clustering of helminth species by infection indices and presence–absence matrices for infracommunities of common fish species from Crimea. (**a**)—*Chelon auratus*; (**b**)—*Mugil cephalus*; (**c**)—*Planiliza haematocheilus*; (**d**)—*Chelon saliens*; (**e**)—*Atherina boyeri*; (**f**)—*Atherina hepsetus*; (**g**)—*Belone belone*; (**h**)—*Diplodus annularis*; (**i**)—*Trachurus mediterraneus*; (**j**)—*Spicara smaris*; (**k**)—*Alosa immaculata*; (**l**)—*Engraulis encrasicolus*. See Table 2 for the parasite species codes.

Dominating (core) species in the different helminth communities ranged from 13 to 67% of their species richness, but in eight of the 12 communities, they accounted for no more than a third of the total number of species (Figure 3 and Figure 5).

There were no significant relationships (Spearman correlation analysis, *p* > 0.05) between the total number of parasite species in fish and the number of core species in the respective communities as well as between the number of specialist species and the composition and number of dominant species (Table 1 and Table 6).

For example, in mullets (golden grey mullet, grey mullet, redlip mullet, and leaping mullet), the core species group was composed of helminths parasitizing only the representatives of this fish family (Table 6).

True specialists (helminths infecting only one host species) such as the monogeneans *Ligophorus mediterranneus* and *L. cephali* were the most common and abundant in the helminth communities of grey mullet as well as *L. pilengas* and *L. llewellyni*—in redlip mullet, and *L. acuminatus*—in leaping mullet. At the same time, the true specialist *Polyclithrum ponticum* were rare in the parasite communities of grey mullet whereas generalist species such as trematode *Haplosplanchnus phachysomus* dominated the communities of some mugilid species belonging to different genera.

Similarly, a specialist monogenean, *Axine belones*, dominated the helminth community of the garfish *Belone belone*, but other dominants were generalist species such as nematode *Hysterothylacium aduncum* and the acanthocephalan *Telosentis exiguous*, parasitizing a wide range of fish species (Table 2). Adult *Hysterothylacium aduncum* were also common and abundant in the helminth communities of the pontic shad *Alosa immaculata* and horse mackerel *Trachurus mediterraneus*, while the larval stages dominated in the communities of the picarel *Spicara smaris* and European anchovy *Engraulis encrasicolus* (Table 6).

There were no relationships between the composition of core species in the communities and their life cycles or developmental stages. For example, all dominating species in the helminth communities of mullets, sea bream, horse mackerel, and pontic shad were parasites reaching maturity in these fishes. In contrast, most core species in the helminth communities of two species of sand smelt, picarel, and European anchovy were at the larval stage. Similarly, parasites with both direct and complex life cycle were frequently observed among the dominating species (Table 6).

Overall, the prevalence and abundance index of dominant parasites in the component communities ranged from 18 to 80% and from 0.6 to 61.5 ind. per fish, respectively (Table 6).

The structure of the helminth component communities, as assessed using the NODF index, demonstrated good accordance with the nestedness structure where the rarest species occurred in the most diverse infracommunities while the poorest infracommunities were composed of a few dominating species (Figure 5). The NODF index varied from 28 to 58, indicating a non-random, nested distribution (Table 5). At the same time, there were no significant correlations between the NODF indices and the average number of species in the infra- and component communities (r-Spearman was 0.2 and 0.5, respectively, *p* > 0.50).

### 3.3. Distribution of Helminths among Infracommunities and Inter-Specific Relationships in Component Communities

Relationships between the log-transformed mean abundances and their log-transformed variances (S^2^) were analyzed for 31 helminth species to reveal their distribution patterns in the infracommunities (Table 7).

The majority of obtained data showed good correspondence with the linear regression models (R^2^ > 0.72), indicating significant levels of the *b*-coefficient (*p* < 0.05), except for *Dicrogaster contracta* and *Axine belones* (R^2^ < 0.50, *p* > 0.05).

More than two-thirds of the studied helminth species (22) had an aggregated distribution with the exponent *b* ranging from 1.4 to 1.8. For three species (*Ligophorus mediterraneus*, *L. cephali*, and *Neochinorhynchus personatus*), the *b*-value reached ≥2, indicating a much higher degree of parasite aggregation. The former two species had high infection indices whereas the latter species had a much lower prevalence and abundance, comparable to many other helminth species (Figure 2, Table 6), indicating that the differences in the distribution were not associated with the different abundances but more likely depended on some external factors. The remaining 10 species had a uniform distribution (0.7 ≤ *b* ≤ 1.35) (Table 7). There were no relationships between the *b*-level and the number of hosts for each helminth (r-Spearman = 0.19) and between the *b*-level and the parasite abundance calculated for all component communities (r-Spearman = 0.37). In general, life-history traits did not affect the *b*-coefficient for the parasites. However, adult *Hysterothylacium aduncum* had a more aggregated distribution than their larvae (Table 7).

Relationships between the log-transformed mean total abundances of all parasite species and their log-transformed variances (S^2^) were analyzed for component communities of helminths from 10 selected fish species to assess the inter-specific co-distribution (Table 8). The results of this analysis indicated a good fitting of the linear regression models (R^2^ > 0.66) and significant *p*-values for the *b*-coefficients.

Seven of the ten analyzed communities had *b*-coefficients close to 2 (i.e., without significant negative inter-specific relationships). In the remaining three communities, *b*-values were not significantly different from 1 (Table 8). Correlations between the average diversity indices for both the infra- and component communities and the degree of community aggregation (*b*-coefficients) were insignificant, the corresponding r-Spearman values were 0.34 and 0.26, *p* > 0.05. These findings confirmed the conclusion that helminths in the component communities had an aggregated distribution. The aggregation degree was higher at the community level in comparison to the individual level.

## 4. Discussion

Generally, the studied infracommunities and component communities of helminths infecting 12 common fish species in the coastal waters of Crimea were characterized by low species diversity. A total of three to 18 helminth species were found in different fish, the Brillouin index for infracommunities ranged from 0.1 to 1.0, and the Shannon index for component communities from 0.3 to 1.2 (Table 3 and Table 4). Some authors believe that low diversity indices of parasite infracommunities and component communities indicate that these should be classified as poor and unbalanced [33,34,98,99]. We found that monogeneans occurred in all of the studied component communities. This result is more likely explained by their direct life cycles and high host specificity (almost half of the monogenean species were specialist parasites).

More diverse helminth communities have been reported by Pankov [69] for mullets off the Bulgarian coast of the Black Sea. *Chelon auratus*, *C. saliens*, *Planiliza haematocheilus*, and *Mugil cephalus* harbored 22, 21, 21, and 20 parasite species, respectively, with HB indices of 0.7, 0.45, 0.4, and 0.25. The values of parasite species diversity in the same fish species off Crimea were 18, 6, 7, and 12, respectively, or 2–3 times lower than in the Bulgarian waters, while the HB values were either lower (in the case of *C. saliens*, 0.2), or comparable (in the case of *C. auratus*, 0.6), or higher (in the cases of *P. haematocheilus*, 0.6 and *M. cephalus*, 1.0). Variations in the Berger–Parker index in both regions were comparable for *C. auratus*, *C. saliens*, and *P. haematocheilus* (0.7–0.9) while the helminth infracommunities in *M. cephalus* from the Crimean waters had a lower value (0.5 vs. 0.9). The substantial differences between the helminth communities of *M. cephalus* off Crimea and Bulgaria may be explained by different proportions of specialist species in the communities compared from these regions (30% vs. 10%). Overall, we observed a positive relationship between the number of specialist species in the analyzed helminth communities of 12 fish species in the Crimean region and their diversity.

In the Mediterranean Sea, common sparid fish species (*Boops boops*, *Spicara maena*, *Pagrus pagrus*, and *Pagellus bogaraveo*) had 26, 26, 24, and 23 helminth species, respectively, a HB index of 0.6–0.8, 0.3–0.7, 0.7, and 0.7, and a Berger-Parker index (d) of 0.5, 0.5–0.8, 0.5, and 0.6, respectively [36,41,100]. The sparid species *Spicara smaris* in the Crimean coastal waters had similar values of HB and d indices, but significantly lower species richness of 6. Thus, despite the substantial difference in species richness, other diversity indices of the helminth infracommunities were comparable in both aquatic areas. These comparisons confirm the opinion that species richness and the diversity of infra- and component communities are uncorrelated with each other [1,101]. This result, however, contradicts some previous data. For example, an analysis of helminth communities of 32 British freshwater fish species described a significant positive correlation between the maximum number of species in a component community and the total number of species in that host species in the region [102]. However, the authors also noted that regional species richness is not a key determinant of species diversity in component communities. Other factors such as the proportion of specialist species in the fauna may influence the diversity of local infra- and component communities.

All component communities of helminths infecting the 12 fish species off Crimea fit the “core–satellite species” model and the dominant species accounted for no more than one-third of the total number of species in most of the analyzed communities (Figure 3 and Figure 4, Table 5). This result is quite consistent with the general principle that, in a particular region, the number of rare species is higher than that of the dominating species [103].

A relationship between the prevalence and log-transformed abundance index that corresponds to a core-satellite species distribution has previously been suggested to be associated with different parasite specificity [21,104] or availability of resources (host fishes) [104]. It assumes that parasites with low host specificity will have a wider occurrence and higher abundance. This hypothesis has been confirmed for *Dactylogyrus* monogeneans [105] but rejected for parasitic nematodes [103]. Our data do not fully match this pattern.

Previous studies considered different infestation indices to delineate dominating, secondary, and rare species of parasites in a community. Some authors have used the prevalence as a metric and set a level of >70% as an indicator of dominating species and a level of <40% as an indicator of secondary or rare species [106,107]. Other authors used an abundance index with the following gradation: core species—>2 ind. per fish, secondary—0.6–2 ind. per fish, satellite—0.2–0.6 ind. per fish, and rare—<0.2 ind. per fish [108]. Bush et al. [109] distinguished parasites according to the following criteria: dominating species parasitize >2/3 of potential hosts with high abundances, secondary species parasitize 1/3–2/3 of potential hosts with medium abundances, and rare species parasitize <1/3 of hosts with low abundances. The respective values of the mean abundance index were set at >30, 20–30, and <30 ind. per host for autogenous species and >75, 50–75, and <50 ind. per fish for allogenous species.

When applying these metrics, we found that only two of the 12 communities (picarel and redlip mullet) had a mean prevalence of dominating species >70% whereas in the remaining communities, these levels were <60%. In addition, nine of the 12 communities demonstrated abundance indices higher than two ind. per fish for core species (Table 6). Thus, the above-mentioned metrics do not fully agree with the true groups of core species. In contrast, the “core-satellite” model, which examines the relationships between the prevalence and log-transformed abundance and the characteristics of parasite occurrences agrees with the true structure, despite the different ranges of abundance indices for the core, satellite, and rare species in each community. Moreover, cluster analyses showed a large degree of overlap between the modal classes and clusters (Figure 3 and Figure 5).

A comprehensive study conducted by Rohde et al. [52] on helminth communities in 102 species of marine teleost fishes from various locations has shown that metazoan ectoparasites live in non-saturated and little-ordered communities and that most parasite species had a distinctly aggregated distribution in their host populations, and prevalence and abundance were positively correlated with each other. They did not find bimodally distributed core and satellite species, probably because of the low sample sizes. Other authors have suggested that the chance for a community to follow the nested structure increases with an increase in the prevalence, abundance, and mean number of species per host [56,57]. Typical Shannon index values vary between 1.5 and 3.5 [110], but in the helminth component communities of the studied Crimean fish, H was on average less than 1.5, and the maximum values observed for most communities did not exceed this level. Thus, in general, these component communities were characterized by a rather low diversity, but were well-ordered communities with a nested structure, as assessed by the NODF index (Table 5). Stable parasite community structures, despite relatively low infection indices in fish hosts, have been also reported for other aquatic systems [35,111,112,113]. Moreover, similar results have recently been reported for two members of the family Sparidae (*Pagrus pagrus* and *Pagellus bogaraveo*) from the western Mediterranean Sea by Lablack et al. [36], who found a homogeneous taxonomic composition of parasite communities for common taxa but not homogeneous for rare taxa (i.e., about the same as took place for *Spicara smaris* in the Crimean waters (Figure 5j)), suggesting a similar use of a common local pool of helminths.

Members of infracommunities, which, in turn, are parts of the component community, can demonstrate a uniform, random, or aggregated distribution and the distribution type can help understand the basic processes in the populations of parasites [32,72,114]. An aggregated distribution of parasites, when only a few host specimens demonstrate heavy parasite burdens while for the majority of individuals the infestation indices are low, is very common in natural populations [115].

Relationships between log-transformed values of S^2^ and log-transformed values of parasite abundances indicated *b*-values of 1–2 (Table 7) in the majority of the analyzed parasite species (21 of 31), thus showing a close resemblance to those reported for other species [96,116,117]. These patterns are in accordance with a defining feature of metazoan parasite populations, which exhibit aggregated distributions among individual hosts [118]. The processes responsible for parasite aggregation involve heterogeneity in the rates at which parasites are acquired or lost from the hosts. Parasite aggregation may result from uneven distributions of infective stages in time and space relative to hosts (i.e., spatial heterogeneity of parasite populations) [112,119,120] and genetic/acquired variation in susceptibility to infection arising from differences among hosts in immune resistance or behavior [121,122]. Some experimental studies have provided evidence that heterogeneity in exposure is more important than heterogeneity in susceptibility (i.e., individual susceptibility of hosts to parasites) [123,124]. The biological sense of such a distribution is explained by the trade-off between very dense aggregations and random distributions. In the first case, the risk to be eliminated is very high due to the increased mortality of too infected hosts, while in the second case, the chance to find a suitable host is low [97]. A recent study conducted by Sarabeev et al. [71,72] for the whole helminth community in the introduced population of the redlip mullet *Planiliza haematocheilus* showed that helminth species distribution in communities from the invasive fish was less aggregated than from its native population, except for monogeneans. The authors concluded that the pattern of parasite aggregation may explain the success of invasive species in ecosystems. Thus, a non-random distribution is considered as a characteristic of stable and well-balanced host–parasite systems [97].

A variety of studies have shown that biotic interactions occur not only between hosts and their symbionts including parasites, but also among symbionts of the same host [125,126,127]. Such interactions can play a role in shaping the community structure of co-occurring species [128,129,130]. During co-infection, helminth species can demonstrate different types of effects on each other, ranging from dramatically positive and null to dramatically negative, depending on the details of their interaction [131]. The *b*-coefficient is an indicator of relationships between different species within the same community: when its value >2, no competition occurs in the community and a decrease in its level indicates negative relationships between the community members [132]. Ma [133] developed the approach of Taylor [96] and extended his model from the population to the community level.

The analysis of the helminth communities in fish off Crimea revealed a higher degree of aggregation for all species (b ≈ 2) when compared to the distribution of individuals of each species. At the same time, such *b*-values in most communities (Table 8), indicate the absence of significant negative relationships between helminths [133] in the analyzed component communities. A great deal of parasitological research has focused on the relative roles of co-infection patterns in driving the composition and structure of parasite communities. Different parasites can induce different immune responses, resulting in either accelerated or delayed negative consequences for co-infected hosts [131]. Research of such synergistic and antagonistic relationships among different parasite species in communities existing in common Crimean fish species where they co-occur is a prospect for further research.

## 5. Conclusions

Our results provide novel data on the structure and diversity of helminth infra- and component communities in fish hosts from coastal waters of the Crimean Peninsula. All of the 12 selected fish species harbored from three to 18 parasites with a total number of 53 helminth species. The highest species richness was registered for golden grey mullet, sand smelt, and grey mullet, while the lowest was for Pontic shad and European anchovy. The most common helminths were host-specific monogeneans of the genus *Ligophorus*. Core and satellite species in the studied component communities were well-distinguishable, as revealed by distribution analysis, which showed bimodality for most cases. Most helminth parasites exhibited an aggregated distribution in the infracommunities. In general, despite low species diversity, the component communities in the region were well-balanced and developed in terms of their nested structures and the aggregated distribution of their members across infracommunities. Further research is necessary to reveal the diversity and structure of parasite communities in other fish species and regions of the Black and Azov Seas and investigate decisive factors responsible for variability in their characteristics.

## Figures and Tables

**Table 1:** List of investigated fish species and information on the examined helminth communities in waters near the Crimean Peninsula during the period 2009–2016.

Trivial Name	Latin Name	Life Style	Number of Communities		Number of Parasite Species			
			Infra-Communities	Component Communities	T	FS	GS	SS
Golden grey mullet	*Chelon auratus*	D	244	15	18	8	3	0
Sand smelt	*Atherina boyeri*	D	448	14	15	0	0	0
Grey mullet	*Mugil cephalus*	D	62	6	12	5	1	3
Garfish	*Belone belone*	P	214	10	9	0	0	2
Redlip mullet	*Planiliza haematocheilus*	D	36	3	7	4	0	2
Leaping mullet	*Chelon saliens*	D	36	3	6	3	2	0
Annular sea bream	*Diplodus annularis*	D	57	4	6	1	1	0
Horse mackerel	*Trachurus mediterraneus*	P	363	17	6	0	0	0
Sand smelt	*Atherina hepsetus*	D	87	5	6	0	0	0
Picarel	*Spicara smaris*	D	129	7	6	0	0	0
Pontic shad	*Alosa immaculata*	A	289	11	5	1	0	0
European anchovy	*Engraulis encrasicolus*	P	246	9	3	0	0	0

Note: D—demersal, P—pelagic, A—anadromous, T—total, FS—family-specific, GS—genus-specific, SS—species-specific.

**Table 2:** Occurrence and infection indices of parasites in helminth component communities of common fish species from waters near the Crimean Peninsula.

Code	Helminth Species	Host Fish Species	CCs × 100/CCt (%)	AI, ind. per Fish, (min ± SE–max ± SE) for CCs
LS	*Ligophorus szidati*	*Chelon auratus*, *Chelon saliens*	78	0.15 ± 0.10–16.00 ± 6.00
LV	*Ligophorus vanbenedenii*	*Chelon auratus*	80	0.06 ± 0.04–72.00 ± 64.00
LA	*Ligophorus acuminatus*	*Chelon auratus*, *Chelon saliens*	17	1.80 ± 1.00–133.00 ± 46.00
LC	*Ligophorus cephali*	*Mugil cephalus*	100	2.80 ± 2.60–144.00 ± 107.00
LM	*Ligophorus mediterraneus*	*Mugil cephalus*	100	1.40 ± 0.70–30.00 ± 7.00
LL	*Ligophorus llewellyni*	*Planiliza haematocheilus*	100	15.00 ± 2.80–20.40 ± 6.8
LiP	*Ligophorus pilengas*	*Planiliza haematocheilus*	100	27.00 ± 3.40–31.00 ± 9.00
LE	*Lamellodiscus elegans*	*Diplodus annularis*	50	2.40 ± 0.50–6.00 ± 2.00
LF	*Lamellodiscus fraternus*	*Diplodus annularis*	25	3.80 ± 1.50
GM	*Gyrodactylus mugili*	*Mugil cephalus*	43	1.30 ± 0.90–8.00 ± 3.50
PP	*Polyclithrum ponticum*	*Mugil cephalus*	14	1.10 ± 0.80
MA	*Mazocraes alosae*	*Alosa immaculata*	90	0.60 ± 0.60–17.00 ± 4.50
SM	*Solostamenides mugilis*	*Planiliza haematocheilus*, *Chelon auratus*, *Mugil cephalus*	56	0.07 ± 0.07–2.60 ± 1.70
AB	*Axine belones*	*Belone belone*	100	0.50 ± 0.30–4.40 ± 1.50
PD	*Progrillotia dasyatidis* L.	*Atherina boyeri*, *Atherina hepsetus*	33	0.03 ± 0.03–0.20 ± 0.10
SP	*Scolex pleuronectis* L.	*Trachurus mediterraneus*, *Belone belone*, *Spicara smaris*	17	0.08 ± 0.06–1.10 ± 0.80
HP	*Haplosplanchnus pachysomus*	*Chelon auratus*, *Mugil cephalus*	83	0.20 ± 0.10–80.00 ± 19.00
DC	*Dicrogaster contracta*	*Chelon auratus*	73	1.00 ± 0.60–9.80 ± 4.00
DP	*Dicrogaster perpusilla*	*Chelon auratus*	40	0.03 ± 0.03–0.40 ± 0.20
SO	*Saccocoelium obesum*	*Chelon auratus*, *Mugil cephalus*	86	0.13 ± 0.13–9.10 ± 4.50
ST	*Saccocoelium tensum*	*Mugil cephalus*, *Chelon auratus*, *Planiliza haematocheilus*, *Chelon saliens*	89	1.10 ± 0.50–30.60 ± 11.00
SS	*Saccocoelium* sp.	*Mugil cephalus*	100	5.60 ± 2.70–100.00 ± 70.00
LeP	*Lecithobotrys putrescens*	*Chelon saliens*	33	0.90 ± 0.60
ShS	*Schikhobalotrema sparisomae*	*Chelon auratus*	67	0.11 ± 0.11–3.00 ± 1.40
AM	*Arnola microcirrus*	*Diplodus annularis*	75	1.00 ± 0.50–2.60 ± 0.90
BB	*Bacciger bacciger*	*Atherina boyeri*, *Atherina hepsetus*	61	0.40 ± 0.30–49.00 ± 13.00
MM	*Monorchis monorchis*	*Diplodus annularis*, *Spicara smaris*	67	0.10 ± 0.08–27.00 ± 3.80
HF	*Helicometra fasciata*	*Diplodus annularis*	25	0.50 ± 0.50
LeF	*Lepocreadium floridanus*	*Trachurus mediterraneus*	88	0.13 ± 0.05–9.00 ± 2.70
PrP	*Prodistomum polonii*	*Trachurus mediterraneus*	94	0.70 ± 0.20–29.00 ± 8.00
PrV	*Pronoprymna ventricosa*	*Alosa immaculata*	90	5.60 ± 2.20–123.00 ± 17.00
ApS	*Aphanurus stossichi*	*Alosa immaculata*	18	0.07 ± 0.04–0.60 ± 0.30
PH	*Pseudobacciger harengulae*	*Engraulis encrasicolus*	22	0.06 ± 0.06–0.07 ± 0.03
GN	*Galactosomum nicolai* mtc.	*Belone belone*	10	29.20 ± 8.50
SC	*Stephanostomum cesticillum* mtc.	*Engraulis encrasicolus*, *Trachurus mediterraneus*	62	0.25 ± 0.20–3.50 ± 0.30
SB	*Stephanostomum bicoronatum* mtc.	*Spicara smaris*	13	1.20 ± 0.40
AS	*Ascocotyle sinoecum* mtc.	*Atherina boyeri*, *Chelon saliens*, *Chelon auratus*, *Mugil cephalus*, *Planiliza haematocheilus*	49	0.30 ± 0.30–26.60 ± 11.00
StP	*Stephanoprora polycestus* mtc.	*Atherina boyeri*	14	13.30 ± 3.30–19.60 ± 1.80
PG	*Pygidiopsis genata* mtc.	*Atherina boyeri*	7	0.60± 0.40
PoA	*Podocotyle atherinae* mtc.	*Atherina boyeri*	7	0.14 ± 0.08
GL	*Galactosomum lacteum* mtc.	*Spicara smaris*	38	6.00 ± 1.60–37.60 ± 8.50
NP	*Neochinorhynchus personatus*	*Mugil cephalus*	83	1.00 ± 0.60–9.80 ± 5.70
AA	*Acanthogyrus adriaticus*	*Chelon auratus*	53	0.03 ± 0.03–2.50 ± 2.00
TE	*Telosentis exiguus*	*Atherina boyeri*, *Atherina hepsetus*, *Belone belone*, *Trachurus mediterraneus*	58	0.04 ± 0.03–12.00 ± 3.00
SH	*Southwellina hispida* L.	*Atherina boyeri*	36	0.02 ± 0.02–0.30 ± 0.10
DM	*Dichelyne minutus*	*Atherina boyeri*	14	0.10 ± 0.07–0.20 ± 0.10
HA	*Hysterothylacium aduncum*	*Alosa immaculata*, *Trachurus mediterraneus*, *Belone belone*	96	0.57 ± 0.14–87.00 ± 11.00
Hal	*Hysterothylacium aduncum* L.	*Engraulis encrasicolus*, *Chelon auratus*, *Atherina boyeri*, *Atherina hepsetus*, *Diplodus annularis*, *Spicara smaris*	62	0.04 ± 0.04–30.00 ± 6.00
CR	*Contracaecum rudolphii* L.	*Atherina boyeri*, *Atherina hepsetus*, *Belone belone*, *Trachurus mediterraneus*, *Mugil cephalus*, *Spicara smaris*	66	0.05 ± 0.04–19.00 ± 13.00
CM	*Contracaecum multipapillatum* L.	*Atherina boyeri*, *Atherina hepsetus*, *Belone belone*	37	0.03 ± 0.03–1.60 ± 0.50
CO	*Cosmocephalus obvelatus* L.	*Atherina boyeri*, *Chelon auratus*, *Planiliza haematocheilus*	10	0.11 ± 0.07–0.23 ± 0.11
PaA	*Paracuaria adunca* L.	*Atherina boyeri*, *Chelon auratus*, *Planiliza haematocheilus*, *Mugil cephalus*, *Belone belone*	15	0.06 ± 0.04–0.60 ± 0.30
EE	*Eustrongylides excisus* L.	*Atherina boyeri*, *Alosa immaculata*	16	0.06 ± 0.04–0.60 ± 0.30
PhP	*Philometra* sp.	*Chelon auratus*	20	0.40 ± 0.30–1.40 ± 0.70

Note: AI—abundance index; Min—minimum, Max—maximum; X—mean, SE—standard error; CCs—component communities where the species was registered; CCt—all component communities where the species can be found (=all component communities associated with fish species which is a host for the helminth).

**Table 3:** Diversity indices in the helminth infracommunities (Min–Max/Mean ± SE) of common fish species in waters near the Crimean Peninsula.

Host Species	N	HB	J	d
*Chelon auratus*	1–10/3.00 ± 0.10	0–1.7/0.60 ± 0.03	0.40–1.00/0.80 ± 0.01	0.20–1.00/0.70 ± 0.02
*Atherina boyeri*	1–5/1.80 ± 0.04	0–1.0/0.20 ± 0.01	0.10–1.00/0.80 ± 0.01	0.30–1.00/0.80 ± 0.01
*Mugil cephalus*	2–7/4.20 ± 0.20	0.4–1.5/1.00 ± 0.04	0.30–1.00/0.80 ± 0.02	0.30–0.90/0.50 ± 0.02
*Belone belone*	1–5/1.80 ± 0.06	0–1.2/0.30 ± 0.02	0.05–1.00/0.80 ± 0.02	0.40–1.00/0.80 ± 0.01
*Planiliza haematocheilus*	1–4/2.50 ± 0.20	0–1.2/0.60 ± 0.06	0.60–1.00/0.80 ± 0.02	0.40–1.00/0.70 ± 0.03
*Chelon saliens*	1–3/1.80 ± 0.10	0–0.7/0.20 ± 0.04	0.08–1.00/0.60 ± 0.01	0.60–1.00/0.90 ± 0.03
*Diplodus annularis*	1–4/1.70 ± 0.10	0–0.9/0.30 ± 0.04	0.15–1.00/0.70 ± 0.04	0.50–1.00/0.90 ± 0.02
*Trachurus mediterraneus*	1–6/2.10 ± 0.05	0–1.2/0.40 ± 0.02	0.20–1.00/0.80 ± 0.01	0.30–1.00/0.80 ± 0.01
*Atherina hepsetus*	1–4/1.80 ± 0.10	0–1.0/0.20 ± 0.03	0.20–1.00/0.70 ± 0.04	0.30–1.00/0.80 ± 0.02
*Spicara smaris*	1–4/1.70 ± 0.07	0–0.9/0.30 ± 0.03	0.20–1.00/0.70 ± 0.03	0.40–1.00/0.90 ± 0.02
*Alosa immaculata*	1–5/2.30 ± 0.07	0–1.3/0.40 ± 0.02	0.03–1.0/0.60 ± 0.02	0.30–1.00/0.80 ± 0.01
*Engraulis encrasicolus*	1–3/1.40 ± 0.03	0–0.7/0.10 ± 0.01	0.10–1.00/0.70 ± 0.03	0.30–1.00/0.90 ± 0.01

Note: N—species richness; HB—Brillouin index; J—Pielou’s evenness; d—Berger–Parker dominance index.

**Table 4:** Diversity indices in the helminth component communities (Min–Max/Mean ± SE) of common fish species from waters near the Crimean Peninsula.

Species	N	H	J	d
*Chelon auratus*	1–14/7.10 ± 0.90	0–2.00/1.20 ± 0.10	0.40–0.90/0.70 ± 0.04	0.30–10/0.50 ± 0.04
*Atherina boyeri*	2–8/5.90 ± 0.50	0.60–1.70/1.10 ± 0.10	0.40–0.90/0.70 ± 0.05	0.30–0.80/0.50 ± 0.05
*Mugil cephalus*	2–10/5.00 ± 0.70	0.20–1.60/1.00 ± 0.10	0.30–1.00/0.70 ± 0.05	0.40–0.90/0.60 ± 0.04
*Belone belone*	1–7/3.70 ± 0.50	0–1.20/0.70 ± 0.10	0.20–0.90/0.60 ± 0.08	0.40–1.00/0.70 ± 0.07
*Planiliza haematocheilus*	1–7/2.90 ± 0.60	0–1.10/0.60 ± 0.10	0.60–0.90/0.70 ± 0.05	0.50–1.00/0.70 ± 0.06
*Chelon saliens*	2–4/3.00 ± 0.30	0.30–0.70/0.40 ± 0.11	0.40–0.60/0.40 ± 0.07	0.70–0.90/0.80 ± 0.06
*Diplodus annularis*	2–4/3.00 ± 0.40	0.30–1.10/0.60 ± 0.20	0.30–0.80/0.50 ± 0.10	0.60–0.90/0.80 ± 0.07
*Trachurus mediterraneus*	2–6/4.50 ± 0.30	0.10–1.30/0.90 ± 0.060	0.20–0.90/0.60 ± 0.04	0.40–0.90/0.60 ± 0.03
*Atherina hepsetus*	4–6/4.80 ± 0.50	0.70–1.10/0.90 ± 0.10	0.40–0.80/0.60 ± 0.10	0.50–0.80/0.70 ± 0.06
*Spicara smaris*	2–5/3.10 ± 0.40	0.05–1/0.60 ± 0.13	0.10–0.90/0.50 ± 0.11	0.50–0.90/0.70 ± 0.06
*Alosa immaculata*	1–6/4.00 ± 0.40	0–0.90/0.50 ± 0.10	0.10–0.80/0.40 ± 0.07	0.60–1.00/0.80 ± 0.04
*Engraulis encrasicolus*	1–3/1.70 ± 0.30	0–0.70/0.30 ± 0.10	0.30–0.90/0.60 ± 0.10	0.60–1.00/0.90 ± 0.05

Note: N—species richness; H—Shannon index; J—Pielou’s evenness index; d—Berger–Parker dominance index.

**Table 5:** Comparison of helminth community structures in common fish species from waters near the Crimean Peninsula.

Host Species	Pearson Correlation logAI vs. P	Number of Species		NODF	z-Score for NODF; *p*-Value
		Infracommunity	Component Community		
*Chelon auratus*	0.72	3	7	37	19; <0.01
*Atherina boyeri*	0.80	2	6	28	26; <0.01
*Mugil cephalus*	0.73	4	5	57	6.3; <0.01
*Belone belone*	0.80	2	4	37	20.6; <0.01
*Planiliza haematocheilus*	0.89	2.5	3	49	1.8; <0.05
*Chelon saliens*	0.90	2	3	61	4; <0.01
*Diplodus annularis*	0.78	2	3	44	5.7; <0.01
*Trachurus mediterraneus*	0.84	2	4.5	53	23; <0.01
*Atherina hepsetus*	0.91	2	5	42	7.3; <0.01
*Spicara smaris*	0.84	2	3	57	19; <0.01
*Alosa immaculata*	0.74	2	4	31	11; <0.01
*Engraulis encrasicolus*	0.87	1.5	2	48	18; <0.01

**Table 6:** Infection indices for parasites in the component communities of common fish species from the coastal waters of the Crimean Peninsula. Mean values are presented with standard errors. Bold font indicates the dominating species.

Host Species	Parasite Species	Prevalence, %	Abundance Index, ind. per Fish
*Trachurus mediterraneus*	*Scolex pleuronectis* L.	2.40 ± 1.30	0.03 ± 0.02
	*Telosentis exiguus*	7.00 ± 5.90	0.70 ± 0.70
	*Contracaecum rudolphii* L.	15.00 ± 3.60	1.40 ± 1.10
	*Lepocreadium floridanus*	15.00 ± 2.80	1.20 ± 0.50
	*Stephanostomum cesticillum* mtc.	19.00 ± 6.90	0.50 ± 0.20
	***Prodistomum polonii***	58.00 ± 6.40	6.00 ± 1.70
	***Hysterothylacium aduncum***	67.00 ± 7.10	5.10 ± 0.90
*Atherina boyeri*	*Eustrongylides excisus* L.	0.14 ± 0.14	0.003 ± 0.003
	*Dichelyne minutus* L.	0.60 ± 0.40	0.010 ± 0.008
	*Pygidiopsis genata* mtc.	0.60 ± 0.60	0.01 ± 0.01
	*Paracuaria adunca* L.	0.90 ± 0.60	0.01 ± 0.005
	*Progrillotia dasyatidis* L.	1.20 ± 0.90	0.02 ± 0.01
	*Cosmocephalus obvelatus* L.	1.60 ± 1.10	0.02 ± 0.01
	*Podocotyle atherinae*	2.10 ± 1.90	0.02 ± 0.01
	*Southwellina hispida*	3.50 ± 1.70	0.04 ± 0.02
	*Contracaecum multipapillatum* L.	6.50 ± 2.20	0.14 ± 0.08
	*Ascocotyle sinoecum* mtc.	11.60 ± 5.20	1.50 ± 1.03
	*Stephanoprora polycestus* mtc.	13.80 ± 9.40	2.30 ± 1.60
	*Hysterothylacium aduncum* L.	15.20 ± 3.60	0.39 ± 0.10
	*Telosentis exiguus*	18.60 ± 5.80	0.79 ± 0.30
	***Bacciger bacciger***	27.10 ± 8.40	9.60 ± 5.40
	***Contracaecum rudolphii* L.**	41.20 ± 6.00	1.20 ± 0.10
*Atherina hepsetus*	*Contracaecum multipapillatum* L.	2.00 ± 1.20	0.02 ± 0.01
	*Progrillotia dasyatidis* L.	10.00 ± 6.50	0.08 ± 0.05
	*Hysterothylacium aduncum* L.	19.50 ± 3.30	0.36 ± 0.10
	***Telosentis exiguus***	30.00 ± 8.90	0.63 ± 0.20
	***Bacciger bacciger***	32.50 ± 18.90	3.93 ± 2.60
	***Contracaecum rudolphii* L.**	39.00 ± 12.00	1.05 ± 0.30
*Diplodus annularis*	*Helicometra fasciata*	1.75 ± 1.75	0.05 ± 0.05
	*Hysterothylacium aduncum* L.	4.75 ± 4.75	0.05 ± 0.05
	*Arnola microcirrus*	20.00 ± 7.00	1.11 ± 0.50
	*Lamellodiscus fraternus*	20.75 ± 7.90	1.04 ± 0.70
	***Lamellodiscus elegans***	42.50 ± 13.10	3.07 ± 0.70
	***Monorchis monorchis***	56.50 ± 9.30	12.02 ± 4.7
*Mugil cephalus*	*Paracuaria adunca* L.	2.00 ± 2.00	0.03 ± 0.03
	*Polyclithrum ponticum*	4.00 ± 4.00	0.47 ± 0.47
	*Solostamenides mugilis*	8.00 ± 4.50	0.6 ± 0.4
	*Contracaecum rudolphii* L.	11.00 ± 6.40	0.28 ± 0.19
	*Gyrodactylus mugili*	19.00 ± 9.20	2.10 ± 1.20
	*Saccocoelium obesum*	24.00 ± 10.10	3.60 ± 1.70
	***Neochinorhynchus personatus***	38.00 ± 11.10	4.49 ± 1.90
	***Saccocoelium tensum***	41.00 ± 9.50	12.82 ± 4.40
	***Saccocoelium* sp.**	52.00 ± 12.80	42.00 ± 11.40
	***Haplosplanchnus pachysomus***	57.00 ± 11.00	32.40 ± 11.30
	***Ligophorus mediterraneus***	69.00 ± 8.90	21.20 ± 7.20
	***Ligophorus cephali***	81.00 ± 5.40	57.00 ± 20.20
*Chelon saliens*	*Haplosplanchnus pachysomus*	5.00 ± 5.00	0.07 ± 0.07
	*Lecithobotrys putrescens*	6.00 ± 6.00	0.30 ± 0.30
	*Saccocoelium tensum*	7.00 ± 6.70	1.20 ± 1.10
	*Ascocotyle sinoecum* mtc.	25.00 ± 25.00	3.20 ± 3.20
	***Ligophorus szidati***	50.00 ± 25.60	5.40 ± 2.90
	***Ligophorus acuminatus***	66.70 ± 33.40	61.50 ± 38.80
*Planiliza haematocheilus*	*Solostamenides mugilis*	4.00 ± 4.00	0.07 ± 0.07
	*Contracaecum rudolphii* L.	7.00 ± 7.00	0.14 ± 0.14
	*Paracuaria adunca* L.	14.70 ± 9.70	0.24 ± 0.14
	*Ascocotyle sinoecum* mtc.	17.70 ± 8.87	2.26 ± 1.15
	*Saccocoelium tensum*	20.30 ± 6.10	1.22 ± 0.50
	***Ligophorus llewellyni***	69.70 ± 10.20	13.50 ± 1.80
	***Ligophorus pilengas***	69.70 ± 10.20	21.60 ± 2.70
*Belone belone*	*Scolex pleuronectis* L.	0.70 ± 0.70	0.01 ± 0.01
	*Cosmocephalus obvelatus* L.	0.90 ± 0.90	0.02 ± 0.02
	*Paracuaria adunca* L.	2.70 ± 2.70	0.03 ± 0.03
	*Galactosomum nicolai*	3.90 ± 3.90	2.60 ± 2.60
	*Contracaecum multipapillatum* L.	4.00 ± 4.00	0.06 ± 0.06
	*Contracaecum rudolphii* L.	12.20 ± 3.00	0.18 ± 0.04
	***Telosentis exiguus***	29.90 ± 10.00	1.47 ± 0.60
	***Hysterothylacium aduncum***	30.10 ± 9.00	1.36 ± 0.60
	***Axine belones***	37.60 ± 2.40	1.19 ± 0.30
*Alosa immaculata*	*Eustrongylides excisus* L.	3.00 ± 1.90	0.06 ± 0.04
	*Aphanurus stossichii*	6.00 ± 3.70	0.17 ± 0.04
	***Mazocraes alosae***	39.30 ± 6.10	3.34 ± 1.40
	***Hysterothylacium aduncum***	44.00 ± 8.10	13.90 ± 7.30
	***Pronoprymna ventricosa***	52.00 ± 8.90	48.60 ± 13.20
*Chelon auratus*	*Paracuaria adunca* L.	0.70 ± 0.70	0.01 ± 0.01
	*Contracaecum rudolphii* L.	0.90 ± 0.60	0.01 ± 0.01
	*Hysterothylacium aduncum* L.	0.90 ± 0.70	0.01 ± 0.009
	*Cosmocephalus obvelatus* L.	0.90 ± 1.00	0.01 ± 0.01
	*Ligophorus acuminatus*	1.40 ± 1.40	0.12 ± 0.10
	*Philometra* sp.	2.40 ± 1.40	0.10 ± 0.05
	*Contracaecum multipapillatum* L.	4.00 ± 1.60	0.04 ± 0.01
	*Acanthogyrus adriaticus*	9.00 ± 3.90	0.50 ± 0.22
	*Dicrogaster perpusilla*	10.00 ± 2.90	0.20 ± 0.06
	*Solostamenides mugilis*	13.70 ± 2.70	0.20 ± 0.06
	*Ascocotyle sinoecum* mtc.	13.90 ± 5.80	1.40 ± 0.90
	*Schikhobalotrema sparisomae*	15.00 ± 4.20	0.80 ± 0.20
	***Haplosplanchnus pachysomus***	26.00 ± 5.20	2.00 ± 0.60
	***Dicrogaster contracta***	32.00 ± 8.40	3.00 ± 0.80
	***Saccocoelium obesum***	33.80 ± 7.50	3.00 ± 0.80
	***Saccocoelium tensum***	39.40 ± 8.20	8.80 ± 2.40
	***Ligophorus szidati***	40.20 ± 6.10	6.80 ± 1.70
	***Ligophorus vanbenedenii***	46.90 ± 6.90	15.90 ± 4.70
*Spicara smaris*	*Scolex pleuronectis* L.	4.60 ± 2.90	0.20 ± 0.16
	*Stephanostomum bicoronatum* mtc.	5.00 ± 5.00	0.20 ± 0.15
	*Contracaecum rudolphii* L.	17.40 ± 5.90	0.50 ± 0.30
	*Monorchis monorchis*	18.00 ± 9.00	1.90 ± 1.10
	*Galactosomum lacteum* mtc.	31.80 ± 15.40	7.10 ± 5.20
	***Hysterothylacium aduncum* L.**	87.80 ± 5.10	9.13 ± 1.80
*Engraulis encrasicolus*	*Pseudobacciger harengulae*	0.90 ± 0.60	0.01 ± 0.008
	***Hysterothylacium aduncum* L.**	36.00 ± 10.70	5.00 ± 2.90
	***Stephanostomum cesticillum* mtc.**	52.60 ± 8.80	1.50 ± 0.40

**Table 7:** Distribution of parasites within the component communities of common fish species in waters near the Crimean Peninsula.

Helminth	Number of Host Species	Number of Component Communities	Equation Parameters		
			*b* ± SE	R^2^	*p*
*Stephanostomum cesticillum* mtc.	2	16	0.7 ± 0.22	0.42	0.007
*Bacciger bacciger*	2	11	1.2 ± 0.12	0.92	<0.001
*Dicrogaster contracta*	1	11	1.2 ± 0.50	0.42	0.030
*Progrillotia dasyatidis* L.	2	6	1.2 ± 0.12	0.96	<0.001
*Pronoprymna ventricosa*	1	10	1.2 ± 0.20	0.83	<0.001
*Southwellina hispida*	1	5	1.2 ± 0.12	0.97	0.002
*Paracuaria adunca* L.	5	7	1.25 ± 0.15	0.93	<0.001
*Axine belones*	1	10	1.35 ± 0.70	0.32	0.090
*Contracaecum rudolphii* L.	6	50	1.4 ± 0.08 *	0.86	<0.001
*Contracaecum multipapillatum* L.	3	15	1.4 ± 0.12 *	0.91	<0.001
*Hysterothylacium aduncum* L.	6	30	1.4 ± 0.10 *	0.86	<0.001
*Acanthogyrus adriaticus*	1	8	1.5 ± 0.13 *	0.96	<0.001
*Dicrogaster perpusilla*	1	6	1.5 ± 0.20	0.90	0.004
*Monorchis monorchis*	2	8	1.5 ± 0.17 *	0.92	<0.001
*Prodistomum polonii*	1	16	1.5 ± 0.11 *	0.93	<0.001
*Saccocoelium obesum*	2	18	1.5 ± 0.15 *	0.86	<0.001
*Telosentis exiguus*	3	26	1.5 ± 0.08 *	0.94	<0.001
*Ligophorus szidati*	2	14	1.55 ± 0.3	0.72	<0.001
*Ligophorus vanbenedenii*	1	12	1.6 ± 0.25 *	0.81	<0.001
*Saccocoelium* sp.	1	6	1.6 ± 0.20 *	0.93	0.001
*Solostamenides mugilis*	3	14	1.6 ± 0.12 *	0.94	<0.001
*Hysterothylacium aduncum*	3	35	1.7 ± 0.10 *	0.90	<0.001
*Ascocotyle sinoecum* mtc.	3	17	1.7 ± 0.12 *	0.93	<0.001
*Haplosplanchnus pachysomus*	2	20	1.7 ± 0.08 *	0.96	<0.001
*Lepocreadium floridanus*	1	15	1.7 ± 0.13 *	0.93	<0.001
*Saccocoelium tensum*	2	24	1.7 ± 0.16 *	0.83	<0.001
*Schikhobalotrema sparisomae*	1	10	1.7 ± 0.20 *	0.90	<0.001
*Mazocraes alosae*	1	10	1.8 ± 0.10 *	0.98	<0.001
*Scolex pleuronectis* L.	2	6	1.8 ± 0.23 *	0.94	0.001
*Ligophorus mediterraneus*	1	7	2.0 ± 0.20 *	0.94	<0.001
*Ligophorus cephali*	1	7	2.3 ± 0.20 *	0.96	<0.001
*Neochinorhynchus personatus*	1	5	2.5 ± 0.13 *	0.99	<0.001

Note: Each component community includes at least 10 fish specimens infected with a given parasite; asterisks indicate *b*-levels significantly different than 1 (*t*-tests, *p* < 0.05).

**Table 8:** Distribution and diversity of parasites within the component communities of fish from Crimea.

Host Species	N1	N2	*HB*	*H*	Equation Parameters		
					*b* ± SE	R^2^	*p*
*Spicara smaris*	6	7	0.3	0.6	1.85 ± 0.44	0.81	0.014
*Chelon auratus*	18	15	0.6	1.2	1.88 ± 0.21 *	0.86	<0.001
*Alosa immaculata*	5	11	0.4	0.5	1.92 ± 0.23 *	0.88	<0.001
*Trachurus mediterraneus*	6	17	0.4	0.9	1.97 ± 0.16 *	0.91	<0.001
*Atherina boyeri*	15	14	0.2	1.1	1.97 ± 0.18 *	0.91	<0.001
*Atherina hepsetus*	6	5	0.2	0.9	2.01 ± 0.27 *	0.96	0.018
*Mugil cephalus*	12	6	1.0	1.0	2.30 ± 0.74	0.66	0.014
*Diplodus annularis*	6	5	0.3	0.6	2.40 ± 0.52	0.92	0.042
*Engraulis encrasicolus*	3	9	0.1	0.3	2.57 ± 0.21 *	0.95	<0.001
*Belone belone*	9	10	0.3	0.7	2.70 ± 0.35 *	0.88	<0.001

Note: N1—Number of helminth species; N2—Number of component communities; *H*—Shannon index; *HB*—Brillouin index. Asterisks indicate *b*-levels significantly different than 1 (*t*-tests, *p* < 0.05).

## Data Availability

All data and sources are presented in the text.
